# Comparison of two computed tomography perfusion post-processing software to assess infarct volume in patients with acute ischemic stroke

**DOI:** 10.3389/fnins.2023.1151823

**Published:** 2023-04-25

**Authors:** Jiayang Liu, Jingjie Wang, Jiajing Wu, Sirun Gu, Yunzhuo Yao, Jing Li, Yongmei Li, Huanhuan Ren, Tianyou Luo

**Affiliations:** ^1^Department of Radiology, The First Affiliated Hospital of Chongqing Medical University, Chongqing, China; ^2^Department of Radiology, Hospital of PLA Army, Chongqing, China; ^3^Department of Radiology, Chongqing General Hospital, Chongqing, China

**Keywords:** acute ischemic stroke (AIS), computed tomography perfusion (CTP), post-processing software, final infarct volume (FIV), ischemic core (IC), penumbra

## Abstract

**Objectives:**

We used two automated software commonly employed in clinical practice—Olea Sphere (Olea) and Shukun-PerfusionGo (PerfusionGo)—to compare the diagnostic utility and volumetric agreement of computed tomography perfusion (CTP)-predicted final infarct volume (FIV) with true FIV in patients with anterior-circulation acute ischemic stroke (AIS).

**Methods:**

In all, 122 patients with anterior-circulation AIS who met the inclusion and exclusion criteria were retrospectively enrolled and divided into two groups: intervention group (*n* = 52) and conservative group (*n* = 70), according to recanalization of blood vessels and clinical outcome (NIHSS) after different treatments. Patients in both groups underwent one-stop 4D-CT angiography (CTA)/CTP, and the raw CTP data were processed on a workstation using Olea and PerfusionGo post-processing software, to calculate and obtain the ischemic core (IC) and hypoperfusion (IC plus penumbra) volumes, hypoperfusion in the conservative group and IC in the intervention group were used to define the predicted FIV. The ITK-SNAP software was used to manually outline and measure true FIV on the follow-up non-enhanced CT or MRI-DWI images. Intraclass correlation coefficients (ICC), Bland–Altman, and Kappa analysis were used to compare the differences in IC and penumbra volumes calculated by the Olea and PerfusionGo software to investigate the relationship between their predicted FIV and true FIV.

**Results:**

The IC and penumbra difference between Olea and PerfusionGo within the same group (*p* < 0.001) was statistically significant. Olea obtained larger IC and smaller penumbra than PerfusionGo. Both software partially overestimated the infarct volume, but Olea significantly overestimated it by a larger percentage. ICC analysis showed that Olea performed better than PerfusionGo (intervention-Olea: ICC 0.633, 95%CI 0.439–0.771; intervention-PerfusionGo: ICC 0.526, 95%CI 0.299–0.696; conservative-Olea: ICC 0.623, 95%CI 0.457–0.747; conservative-PerfusionGo: ICC 0.507, 95%CI 0.312–0.662). Olea and PerfusionGo had the same capacity in accurately diagnosing and classifying patients with infarct volume <70 ml.

**Conclusion:**

Both software had differences in the evaluation of the IC and penumbra. Olea’s predicted FIV was more closely correlated with the true FIV than PerfusionGo’s prediction. Accurate assessment of infarction on CTP post-processing software remains challenging. Our results may have important practice implications for the clinical use of perfusion post-processing software.

## 1. Introduction

For patients with acute ischemic stroke (AIS) caused by anterior-circulation large vessel occlusion (LVO), appropriate treatments will significantly improve clinical outcomes (Berkhemer et al., 2015; Demchuk et al., 2015; LeCouffe et al., 2021). The extent of ischemic damage is an important guide for clinical evaluation of treatment risk and benefit. Therefore, accurate and timely assessment of the baseline ischemic core (IC) and penumbra (salvageable tissue in the hypoperfusion area) is essential for proper clinical treatment decisions in patients with AIS (Albers et al., 2018; Nogueira et al., 2018). Patients are likely to benefit the most from appropriate treatment selection based on a precise assessment of infarct status (Ribo et al., 2015).

The success of this estimate is closely linked to the use of appropriate imaging methods to screen patients. Rapid, noninvasive, one-stop multimodality computed tomography (CT) imaging is the guideline-recommended screening modality for AIS (Powers et al., 2019), because it provides better identification of the stroke mechanism than other imaging modalities (Bill et al., 2017). Computed tomography perfusion (CTP) is more accurate at identifying the extent of IC and penumbra and at predicting the final clinical outcome than non-contrast CT (NCCT) and computed tomography angiography (CTA; Parsons et al., 2005; van Seeters et al., 2016). Perfusion post-processing software algorithms are continuously updated to improve computational accuracy and are widely used in clinical practice (Xiong et al., 2019). However, the predicted final infarct volume (FIV) calculated by CTP often differs from the true FIV owing to differences in equipment, scanning parameters, post-processing algorithms, individual patient conditions, and treatments.

Although infarct volume is the main basis for assessing the risk of patient treatment, there are currently no uniform criteria to determine the extent of infarction (Campbell et al., 2015; Vagal et al., 2019). Different software have different algorithms and use different thresholds of measurement (Sakai et al., 2018; Nael et al., 2019; Rava et al., 2021a). In clinical practice, we found that different IC, penumbra, and predicted FIV were obtained for the same patient with different software analyses, which confused clinicians and made decision-making more challenging. Therefore, this study aimed to analyze the difference between the Olea Sphere [Olea; V3.0 SP28 (Olea Medical, La Ciotat, France)] and Shukun-PerfusionGo [PerfusionGo; V2.4 (Digital Kun Technology Co. Ltd., Beijing, China)] software packages, currently widely used in China, to evaluate the volume of acute IC and penumbra and compare the volumetric consistency of predicted FIV and true FIV between the two CTP software programs.

## 2. Materials and methods

### 2.1. Study design and patient selection

This study was approved by the institutional review board and ethics committee of The First Affiliated Hospital of Chongqing Medical University, and each patient or an appropriate family member signed the informed consent form.

We collected 122 patients who met the following inclusion criteria between July 2019 and December 2021: (1) clinical diagnosis of AIS and underwent baseline NCCT, CTP, and CTA imaging examination before treatment; (2) occlusion of intracranial anterior-circulation large vessels (terminal internal carotid artery, M1 or M2 segment of the middle cerebral artery) confirmed by CTA; (3) had FIV measured on subsequent CT or diffusion-weighted imaging (DWI) during hospitalization; and (4) patients who underwent acute recanalization treatments [intravenous thrombolysis (IVT) and/or endovascular thrombectomy (EVT)] were successfully reperfused. The exclusion criteria were as follows: (1) history of previous stroke episode; (2) simultaneous existence of neuroimaging findings showing posterior circulation stroke or bilateral infarcts; (3) hemorrhagic transformation hindering the evaluation of imaging; and (4) image quality insufficient for analysis.

We recorded demographic data, medical history, vascular risk factors, treatments, neurologic symptoms and signs, biochemical parameters, and stroke severity based on the National Institutes of Health Stroke Scale [NIHSS] score (range, 0–42, with higher scores indicating more serious neurologic deficits) at baseline, after 24 h, and at 5–7 days or at discharge if earlier. Individual treatment decisions were made according to international guidelines.

### 2.2. Grouping criteria for patients

Patients were divided into two groups (intervention group and conservative group) based on their recanalization of blood vessels and clinical outcome after different treatments. Intervention patients were successfully reperfused after revascularization (mTICI = 2b-3, or a decrease of NIHSS score to 1 or 0, or a total decrease by ≥4 points by the end of the first 24 h after IVT) whereas conservative patients did not receive recanalization treatment and had no significant improvement or even worsening of symptoms after treatment. Because of the significant association between the NIHSS score and the presence and location of vascular occlusion, patients with a continuous decrease in NIHSS score were defined as having significant symptomatic improvement (Yoshimura et al., 2022), conservative patients’ NIHSS score remained the same or increased.

### 2.3. Multimodal CT imaging acquisitions

Patients with suspected AIS underwent a one-stop CT multimodal scan (including NCCT, 4D-CTA/CTP) on a 320-row detector CT scanner (Aquilion ONE, Canon Medical Systems Corporation, Otawara, Japan). NCCT was performed in dynamic volume single-scan mode first, followed by CTA and CTP in whole-brain dynamic volume interval mode from the aortic arch to the top of the frontal sinuses, per the following parameters: 80 kV, 150–310 mA, coverage of 140–160 mm, reconstruction with adaptive iterative dose reduction, 1.0-mm slice thickness, and 1.0-mm interval to improve reconstructive speed. Dynamic volume perfusion scans were started at the 7th second after intravenous injection of iodinated contrast material, with contrast infusion and scanning proceeding simultaneously. Arterial intermittent scans and venous intermittent scans were performed for 2 s and 5 s, respectively, with a total scan time of about 60 s according to the perfusion curves to obtain NCCT and 4D-CTA/CTP data. Injection of an iodine 400 contrast agent (Iopamidol, Bracco Sine, Italy) protocol was implemented using high-pressure injector P3T technology (MEDRAD Stellant CT Injection System, Bayer Medical Care, USA); the said protocol for contrast-agent injection was based on the patient’s sex, weight, height, and intravascular iodinated contrast concentration to automatically calculate the quantity and speed of contrast agent.

### 2.4. Automatic software analysis of CTP data

All patients’ CTP data were transferred to post-processing using the Olea and PerfusionGo software (described earlier), and the IC and hypoperfusion volumes were quantified. The CTP analysis was carried out by using data from both hemispheres, while the lesion side was automatically characterized with a longer time to drain and the contralateral side was used as a reference for relative values. In addition, based on the arterial perfusion curves, we also critically reviewed the imaging results calculated separately by the software for regions with obvious miscalculations (calculated infarct areas in the ventricles and bone). We performed the recommended workflow, including manual checks, adjustments, and correction of the stroke area throughout the perfusion volume.

In the Olea software, hypoperfusion volume was defined by time to peak (TTP) >5 s, and IC was defined as a decrease of cerebral blood flow (CBF) by more than 25% compared to the normal hemisphere. In the PerfusionGo software, hypoperfusion volume was defined by Tmax>6 s, while IC volume was defined as a 30% reduction in CBF compared to the contralateral side.

### 2.5. FIV measurement

Follow-up DWI or NCCT images were acquired to evaluate true FIV. In the intervention group, follow-up imaging was performed within 24 h of recanalization treatment, and patients in the conservative treatment group underwent follow-up imaging within 7 days of treatment. The true FIV was manually outlined independently by two neuroradiologists blinded to the clinical information and with 5 years’ experience by using ITK-SNAP (version 3.8.0). Either the NCCT or the DWI images from patient follow-up were used; DWI was preferred if both were available. DWI provided accurate representations of the infarct by locating regions of restricted diffusion (Rava et al., 2020), so the true final infarct was defined as an area of high signal on T2 weighted fluid attend weighted inversion recovery (T2-FLAIR) and DWI images and reduced signal on ADC images (Kremenova et al., 2022). On NCCT, the area with reduced density was considered the true final infarct area (Wouters et al., 2022). A total of 87 patients had true FIV determined by DWI, and 35 patients had true FIV determined by NCCT. Consecutive layers were outlined on NCCT/DWI axial images of the head, and the infarct area was obtained by multiplying each manually outlined infarct area by the layer thickness volume per layer, and the volumes of each layer were added to obtain the true FIV.

Previous studies have reported that in patients without recombinant tissue plasminogen activator (rt-PA) or thrombectomy, the volume of hypoperfusion is highly correlated with MRI infarct volume (Rao et al., 2019; Xiong et al., 2019). Therefore, to get the predicted FIV from baseline CTP data, we used baseline hypoperfusion volume to predict for patients in the conservative group (Chavez et al., 2009; Rava et al., 2021b). For patients in the intervention group, the predicted FIV was considered to correspond to the IC (Liu et al., 2021).

### 2.6. Statistical analysis

Statistical analysis was performed using SPSS version 22.0 (IBM Corporation, Armonk, NY, USA). The patients’ continuous variable characteristics were presented as the mean standard deviation (SD) or median and interquartile range (IQR). Categorical and binary variables were presented as the absolute frequencies (percentage). Kolmogorov–Smirnov test was used to evaluate normal distributions. Patient characteristics were compared between the intervention group and conservative group using an independent-samples *t*-test.

Wilcoxon’s signed rank test (for skewed data) and paired sample *t*-test (for normally distributed data) were used to compare volumetric differences in the IC and penumbra volumes between different post-processing software and true FIV. Intraclass correlation coefficients (ICC; absolute consistent two-way random model, single measure) and Bland–Altman analysis (95% limits of agreement) were used to identify the correlations between the predicted FIV of Olea/PerfusionGo and true FIV, respectively. In addition, Spearman’s correlation coefficient was used to assess the correlation strength of Olea/PerfusionGo volumes and true FIV. *p* < 0.05 was considered to indicate statistically significant results. To analyze whether Olea and PerfusionGo could accurately classify patients based on the predicted FIV, we classified the subgroups according to whether the true FIV was <70 ml. The diagnostic agreement for a volume < 70 ml between the predicted FIV of Olea/PerfusionGo and the true FIV was determined by kappa values. Kappa values were interpreted according to proposed standards: 0–0.20 slight, 0.21–0.40 fair, 0.41–0.60 moderate, 0.61–0.80 substantial, and 0.81–1.0 perfect.

## 3. Results

### 3.1. Patient characteristics

**Figure 1:**
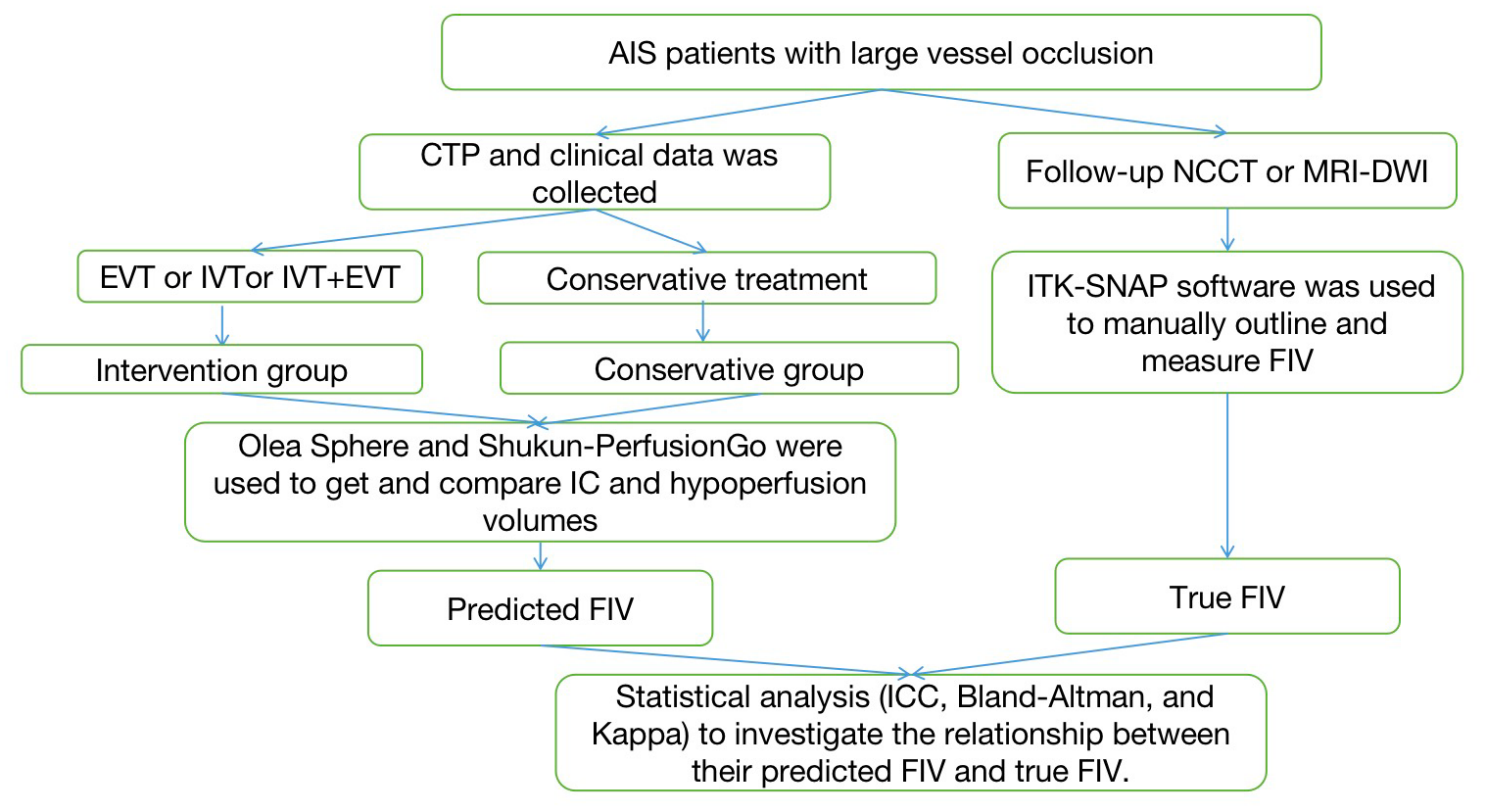
The flowchart of the proposed approach. AIS, acute ischemic stroke; NCCT, non-contrast CT; IVT, intravenous thrombolysis; EVT, endovascular thrombectomy; FIV, final infarct volume.

Patients’ clinical and demographic characteristics are shown in Table 1 along with analysis after separation into the intervention and conservative groups. Fifty-two patients (31 men, 21 women) who achieved successful recanalization were enrolled in the intervention group, and the rest (45 men, 25 women) were included in the conservative group. The mean age was 66.61 ± 13.66 years. The time from onset to perfusion imaging and the time from perfusion imaging to follow-up imaging showed significant differences (*p* < 0.05) between the two groups (38 [73%] and 36[51%] patients were scanned within 24 h after onset in the intervention group and conservative group, respectively). The median initial NIHSS score was 9 (IQR, 2–15), and the median NIHSS score after treatment was 7 (IQR, 2–14). Hypertension was the most common risk factor (65.6%), followed by smoking (41.8%; Figure 1).

**Table 1:** Baseline demographics and characteristics of all enrolled patients.

Characteristics	All (*n* = 122)	Intervention group (*n* = 52)	Conservative group (*n* = 70)
Age (y), (mean ± SD)	66.61 ± 13.66	65.88 ± 15.54	67.14 ± 12.18
Male (%)	76 (62.3)	31 (59.6)	45 (64.3)
Initial NHISS score, median (IQR)	9 (2–15)	9 (3–15)	8 (2–15)
NIHSS score after treatment, median (IQR)	7 (2–14)	6 (0–9)	8 (3–17)
Smoking (%)	51 (41.8)	20 (38.5)	31 (44.3)
Diabetes (%)	40 (32.8)	18 (34.6)	22 (31.4)
Hypertension (%)	80 (65.6)	31 (59.6)	49 (70)
Coronary heart disease (%)	16 (13.1)	7 (13.5)	9 (12.9)
Atrial fibrillation (%)	26 (21.3)	17 (32.7)	9 (12.9)
Hyperlipidemia (%)	46 (37.7)	14 (26.9)	32 (45.7)
*Time from onset to perfusion imaging (%)*
<4.5 h	42 (34.4)	30 (57.7)	12 (17.1)
4.5–6 h	7 (5.7)	3 (5.8)	4 (5.7)
6–24 h	25 (20.5)	5 (9.6)	20 (28.6)
>24 h	48 (39.3)	14 (26.9)	34 (48.6)
Time from perfusion imaging to follow-up imaging (d), (median) (IQR)	2 (0–4)	0 (0–1)	4 (3–6)

NIHSS, national institutes of health stroke scale.

### 3.2. IC and hypoperfusion volume calculated by Olea and PerfusionGo

**Figure 2:**
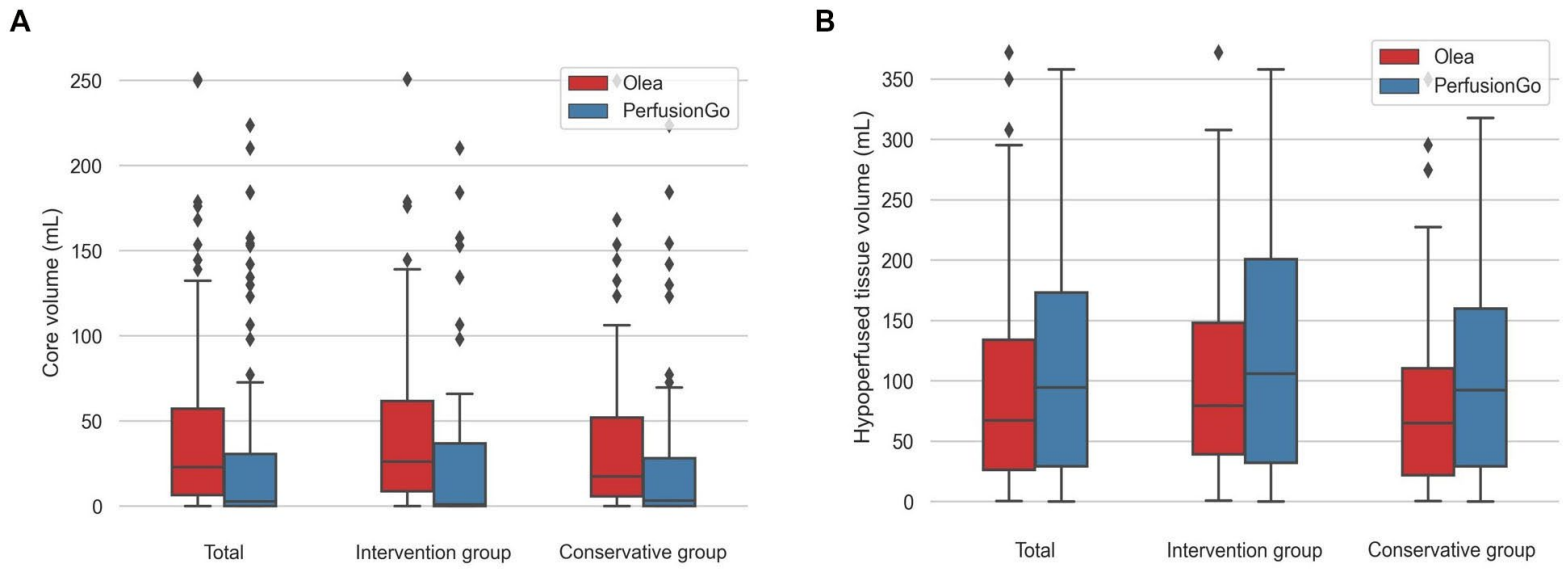
Boxplots of core and hypoperfusion volume estimated by Olea and PerfusionGo software. Bars indicate the range of core and hypoperfusion volume. Boxes represent values within the 25–75th percentiles [interquartile range (IQR)], and horizontal lines represent the median. **(A)** core volume. **(B)** hypoperfusion volume.

The volume of IC and hypoperfusion in all patients are shown in Figure 2 and Table 2, and separate analyses were also performed for the two groups. In terms of all patients’ IC, the median (IQR) volume was 23.38 ml (6.49–57.59) with Olea and 2.90 ml (0.00–30.23) with PerfusionGo. After grouping, median IC volumes were 26.06 ml (8.73–61.79), 0.95 ml (0.00–36.85), 17.39 ml (5.82–52.13), and 3.25 ml (0.00–28.10) for intervention group-Olea, intervention group-PerfusionGo, conservative group-Olea, and conservative group-PerfusionGo, respectively. The IC difference between Olea and PerfusionGo within the same group was statistically significant (*p* < 0.001). The calculated volume of IC with Olea was greater than that with PerfusionGo.

**Table 2:** Ischemic core (IC) and hypoperfusion volume estimated by the Olea and PerfusionGo software.

	Olea			PerfusionGo		
	All	Intervention group	Conservative group	All	Intervention group	Conservative group
Ischemic core (IC), median (IQR), ml	23.38 (6.49–57.59)	26.06 (8.73–61.79)	17.39 (5.82–52.13)	2.90 (0.00–30.23)	0.95 (0.00–36.85)	3.25 (0.00–28.10)
Hypoperfusion volume, median (IQR), ml	67.06 (26.12–134.11)	79.32 (39.08–148.05)	64.90 (21.70–110.35)	94.45 (29.33–173.28)	105.95 (32.35–200.75)	92.25 (29.33–159.83)

Meanwhile, the median (IQR) hypoperfusion volume was 67.06 ml (26.12–134.11) with Olea and 94.45 ml (29.33–173.28) with PerfusionGo. In the two groups, the median volumes were 79.32 ml (39.08–148.05), 105.95 ml (32.35–200.75), 64.90 ml (21.70–110.35), and 92.25 ml (29.33–159.83) for intervention group-Olea, intervention group-PerfusionGo, conservative group-Olea, and conservative group-PerfusionGo, respectively. The difference was statistically significant within the same group (*p* < 0.001). The PerfusionGo calculated penumbra value was larger than that calculated by Olea.

### 3.3. Overestimation of predicted infarct volumes by both software compared with true FIV

**Figure 3:**
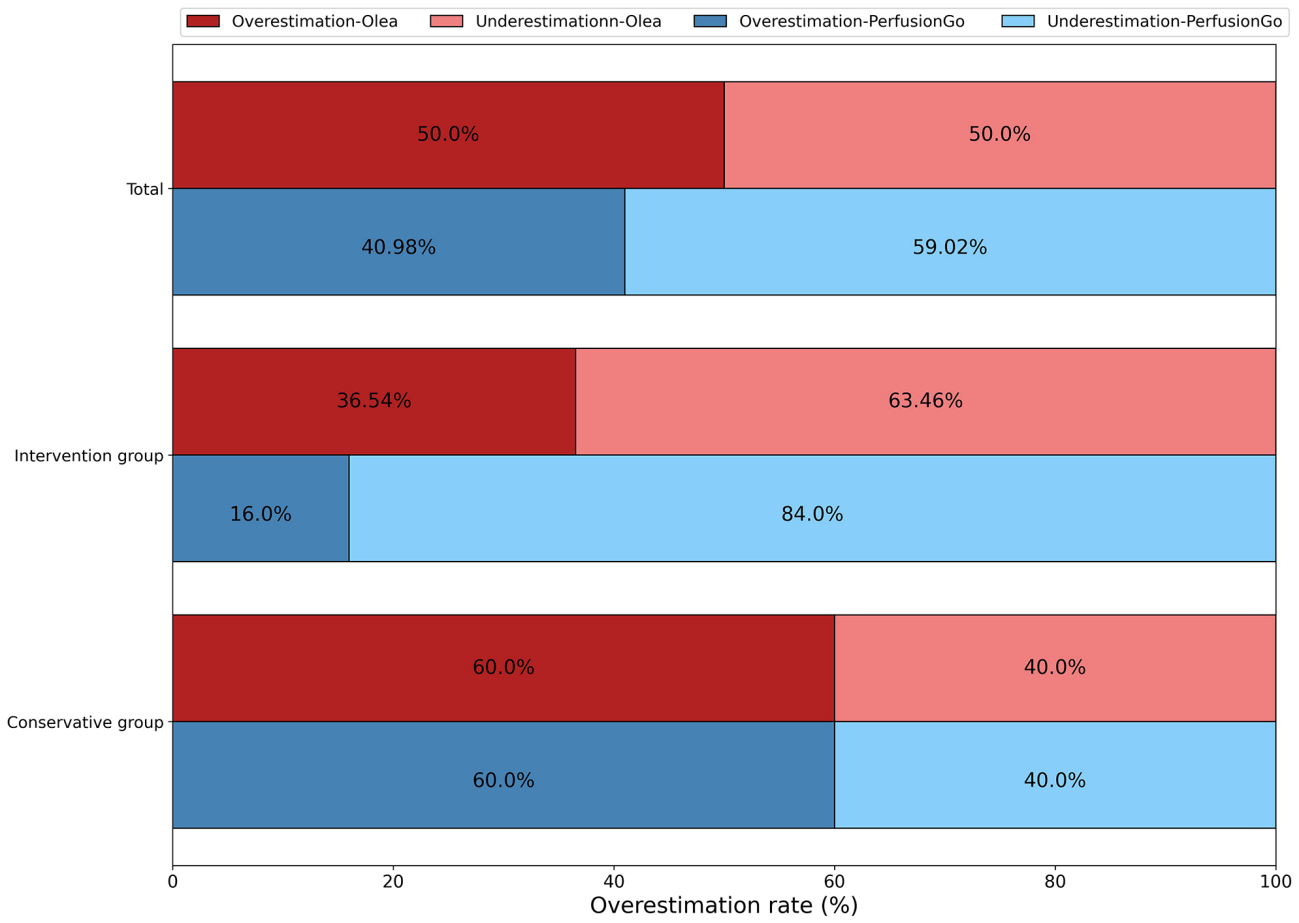
Overestimation of infarct volume by both software as compared with the true final infarct volume (FIV).

In Figure 3, we compared the overestimates of two programs in the same group (total, intervention group, and conservative group). Although both software partially overestimated the infarct volume, we saw that Olea significantly overestimated it by a larger percentage than PerfusionGo. Furthermore, using the same software to compare the overestimates of different groups, patients in the conservative group were more likely to be overestimated than those in the intervention group.

### 3.4. Volumetric correlation of true FIV with predicted FIV

Table 3 shows the correlation between the predicted CTP infarct volumes and DWI-MRI infarct volume in patients with DWI-MRI infarcts. The true FIV was quantified in all patients; the median (IQR) FIV was 34.97 ml (10.56–116.58) in all patients, 26.23 ml (11.15–85.24) in the intervention group, and 39.10 ml (10.28–128.88) in the conservative group. The median (IQR) volumes of the predicted FIV were 26.06 ml (8.73–61.79), 0.95 ml (0.00–36.85), 64.80 ml (21.7–110.35), and 92.25 ml (29.33–159.83) in the intervention group-Olea, intervention group-PerfusionGo, conservative group-Olea, and conservative group-PerfusionGo, respectively.

**Table 3:** Correlation between the predicted final infarct volume (FIV) from Olea/PerfusionGo and true FIV from follow-up DWI/NCCT.

		Follow-up DWI or NCCT	Olea	Shukun-PerfusionGo
Total	Median (IQR) FIV, mL	34.97 (10.56–116.58)	43.15 (14.13–84.80)	42.15 (0.93–111.29)
	Mean (SD) difference in FIV, mL		11.89 (70.03)	5.03 (83.82)
	Limits of agreement FIV, mL		(−125.36–149.14)	(−159.25–169.31)
	Patients with infarct overestimation (%)		50 (61/122)	40.98 (50/122)
	ICC (95%CI)		0.629 (0.509–0.725)	0.535 (0.396–0.650)
	Spearman’s correlation		0.67	0.555
			<0.001	<0.001
Intervention group (*n* = 52)	Median (IQR) FIV, mL	26.23 (11.15–85.24)	26.06 (8.73–61.79)	0.95 (0.00–36.85)
	Mean (SD) difference in FIV, mL		29.58 (66.64)	46.87 (69.80)
	Limits of agreement FIV, mL		(−101.05–160.2)	(−89.94–183.68)
	Patients with infarct overestimation (%)		36.54 (19/52)	15.38 (8/52)
	ICC (95%CI)		0.633 (0.439–0.771)	0.526 (0.299–0.696)
	Spearman’s correlation		0.626	0.621
			<0.001	<0.001
Conservative group (*n* = 70)	Median (IQR) FIV, mL	39.10 (10.28–128.88)	64.80 (21.7–110.35)	92.25 (29.33–159.83)
	Mean (SD) difference in FIV, mL		−1.25 (69.58)	−26.05 (79.74)
	Limits of agreement FIV, mL		(−137.63–135.13)	(−182.34–130.24)
	Patients with infarct overestimation (%)		60 (42/70)	60 (42/70)
	ICC (95%CI)		0.623 (0.457–0.747)	0.507 (0.312–0.662)
	Spearman’s correlation		0.673	0.56
			<0.001	<0.001

**Figure 4:**
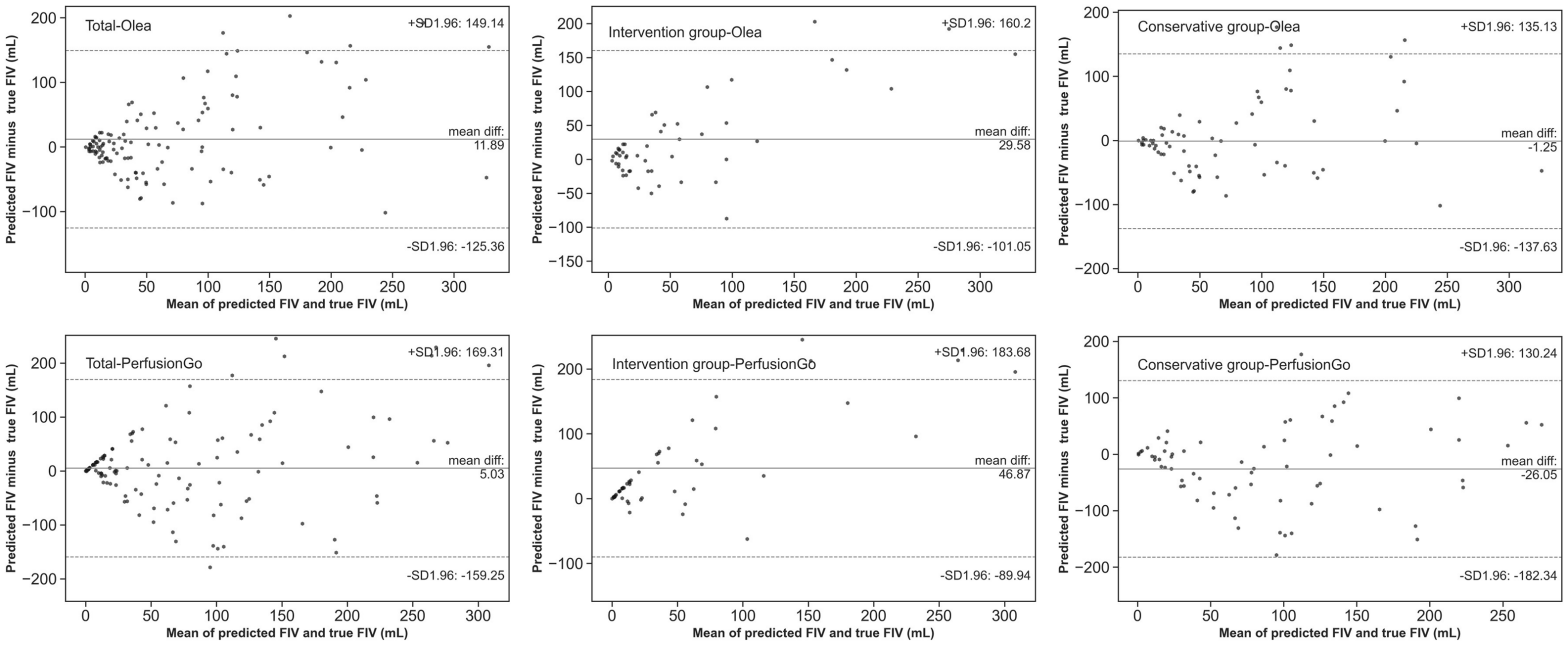
Agreement between true final infarct volume (FIV) and predicted FIV of Olea/PerfusionGo. Solid lines demonstrate the mean difference from FIV. Dotted lines represent 95% limits of agreement.

The Bland–Altman and Spearman’s correlation analyses for each software package against true FIV are shown in Table 3 and Figure 4. Bland–Altman analysis indicated that the mean (SD) difference between the predicted FIV and true FIV was 29.58 (66.64) ml, 46.87 (69.80) ml, −1.25 (69.58) ml, and − 26.05(79.74) ml for the intervention group-Olea, intervention group-PerfusionGo, conservative group-Olea, and conservative group-PerfusionGo, respectively. Total-Olea (ICC 0.629, 95%CI 0.509 to 0.725) showed better results than total-PerfusionGo (ICC 0.535, 95%CI 0.396 to 0.605) in ICC analysis of agreement compared with true FIV. In the subgroup group analysis, the ICC analysis showed that Olea still performed better than PerfusionGo (intervention group-Olea: ICC 0.633, 95%CI 0.439 to 0.771, intervention group-PerfusionGo: ICC 0.526, 95%CI 0.299 to 0.696, conservative group-Olea: ICC 0.623, 95%CI 0.457 to 0.747, and conservative group-PerfusionGo: ICC 0.507, 95%CI 0.312 to 0.662; Table 3). The combined results of ICC, Spearman’s correlation, and Bland–Altman analysis revealed that the Olea-predicted FIV more closely correlated with the true FIV than the PerfusionGo-predicted FIV in all infarct cases; moreover, Olea performed better than PerfusionGo in every group analysis.

### 3.5. Diagnostic agreement for the predictable FIV <70 ml as estimated by true FIV

Olea and PerfusionGo showed substantial agreement with true FIV (Olea: Kappa = 0.47, PerfusionGo: Kappa = 0.5). In the intervention group, PerfusionGo had a slightly better sum of moderate agreement with true FIV (kappa = 0.49) than Olea (kappa = 0.42), but the values were similar in the conservative group (kappa = 0.49; Table 4).

**Table 4:** Accuracy of diagnosing predicted final infarct volume (FIV) of <70 ml as defined on true FIV.

Total				
Baseline CT	DWI infarct volume (FIV) < 70 ml		Kappa	value
	Yes (*n* = 78)	No (*n* = 44)		
Olea
<70 ml	66 (84.62%)	17 (38.64%)	0.47	<0.001
>70 ml	12 (15.38%)	27 (61.36%)		
Shukun-PerfusionGo
<70 ml	60 (76.92%)	11 (25.00%)	0.5	<0.001
>70 ml	18 (23.08%)	33 (75.00%)		
Intervention group (*n* = 52)
Baseline CT	DWI infarct volume (FIV) < 70 ml		Kappa	Value
	Yes (*n* = 35)	No (*n* = 17)		
Olea
<70 ml	32 (91.43%)	9 (52.94%)	0.42	<0.001
>70 ml	3 (8.57%)	8 (47.06%)		
Shukun-PerfusionGo
<70 ml	32 (100.00%)	10 (58.82%)	0.49	<0.001
>70 ml	0 (0.00%)	7 (41.18%)		
Conservative group (*n* = 70)
Baseline CT	DWI infarct volume (FIV) < 70 ml		Kappa	Value
	Yes (*n* = 43)	No (*n* = 27)		
Olea
<70 ml	34 (79.07%)	8 (29.63%)	0.49	<0.001
>70 ml	9 (20.93%)	19 (70.37%)		
Shukun-PerfusionGo
<70 ml	25 (58.14%)	1 (3.70%)	0.49	<0.001
>70 ml	18 (41.86%)	26 (96.30%)		

## 4. Discussion

In this study, we provided an analysis of the differences in IC and penumbra estimated by two commercial CTP post-processing software packages that are widely used in clinical practice in China and compared the volumetric consistency of predicted FIV and true FIV between the CTP software programs. Based on baseline CTP, a larger IC and smaller penumbra was calculated using Olea than PerfusionGo. Although we found that a higher proportion of patients were overestimated by Olea, the results of our study showed better agreement of Olea’s predicted FIV with the true FIV in assessing final infarction, i.e., closer volume correlation and less volume variation than with PerfusionGo. Furthermore, both software showed similar ability to accurately diagnose infarcts volumes <70 ml.

Baseline CTP (Hoving et al., 2018) to assess ischemic damage is the most common method recommended by the guide in the clinical management of AIS patients, given its rapidity and ease (Powers et al., 2019). Accurate quantification of the IC and penumbra in the acute phase is critical, because the volume of the tissue that can be saved and the tissue that currently identifies the infarct directly affects appropriate treatment decisions and clinical outcomes (Gasparotti et al., 2009; Albers et al., 2017; Broocks et al., 2021; Chen et al., 2021). However, discrepancies between the predicted infarct volume and true FIV are prevalent, and accurate assessment of IC and penumbra remain challenging (Schaefer et al., 2015; Albers et al., 2016; Koopman et al., 2019; Chen et al., 2021). Inaccurate assessment can be detrimental to patient health and overall outcome. For example, overestimation of infarction may result in patients not being eligible for reperfusion treatments, while underestimation may increase hemorrhage after reperfusion leading to poor clinical outcomes (Rava et al., 2020). Therefore, we believe that our study will help clinicians to more clearly understand the existence of these differences and make appropriate decisions.

Regarding the IC and hypoperfusion volume analysis, our results showed a volumetric trend, in that the calculated volume of IC by Olea was larger than that calculated by PerfusionGo. The penumbra calculated by PerfusionGo was larger than that calculated by Olea. The same trend could be observed in individual groups. However, there is no uniform standard yet available. This study used the recommended setting of IC and hypoperfusion volume. Based on past research, the IC and penumbra volume measured from CTP were influenced by many factors such as CT scanning parameters (voltage, scanning time); motion correction; and transmission speed of contrast (Kudo et al., 2010; Sotoudeh et al., 2019; Vagal et al., 2019). The advantage of our study is that we used the same scanning equipment and CTP data for all patients in the study to eliminate interference from confounding factors.

Different perfusion parameters and thresholds are used by Olea and PerfusionGo, and previous studies have proven that using varied settings will produce very variable results (Bivard et al., 2013; Austein et al., 2016; Kremenova et al., 2022). This is consistent with our experimental results. These results suggest that determining the optimal method for predicting infarct volume with several different parameters warrants further investigation.

In our study, although both software overestimated the infarct volume, Olea was more likely to overestimate than PerfusionGo, particularly in the conservative group. Overestimation and underestimation of volume have a crucial impact on the choice of treatment modality and prognosis prediction (Horsch et al., 2015; Boned et al., 2017). Although this is still a point of contention, large IC are perceived as a factor of poor outcome, a risk factor for hemorrhagic transformation, and can be used as an exclusion criterion in EVT. If we use PerfusionGo for calculation and prediction, more patients will be eligible for EVT to recover lost neurological function than if we use Olea.

Our results showed that there is a discrepancy between the volume calculated by the software and the final volume. These differences between the predicted FIV and true FIV are likely because of the evolution of infarct following onset which continues to occur between initial estimation, treatment procedures, and followup imaging. Some patients’ conditions worsened after CTP and the penumbra tissue became severely hypoxic. Understandably, not all of the ischemic penumbra calculated from CTP can be salvaged (i.e., TICI = 2b), as an increase in infarct volume may occur during this period, and any detected penumbra may show up as an infarct on follow-up imaging (Guenego et al., 2018). It has been shown that despite successful reperfusion with EVT, considerable infarct growth remains (Bala et al., 2021). The assumption that all penumbra in conservatively treated patients becomes infarcted is a limitation because of the effect of spontaneous recanalization, given that emboli may rupture in some patients, leading to reperfusion without intervention and restoration of the penumbra (Guadagno et al., 2008; Zhang et al., 2023). Additionally, the large variability in treatment outcomes between patients leads to large differences in the transformation of ischemic penumbra.

Olea had a closer volumetric correlation and smaller limits of agreements with true FIV than PerfusionGo, it is better than existing studies (Liu et al., 2021). For the conservative group, this advantage is more obvious. It can be attributed to the fact that Bayesian post-processing algorithms have been used by Olea Sphere CTP software to segment ischemic tissue. Bayesian-based approach showed great reduction in the variability of the estimated IC. However, volume-consistent results appear to be slightly higher in intervention patients than in conservative patients, as these patients have smaller infarct volumes and a greater likelihood of salvage.

The predicted FIV of 70 ml was a criterion for the extent of infarction to help decision-making for IVT and EVT (Bivard et al., 2015). To make therapeutic decisions for borderline cases, e.g., in patients with an infarct ≤70 ml who may be more likely to benefit from reperfusion therapy, we evaluated the diagnostic agreement for the detection of infarct volumes <70 ml as estimated by true FIV. Both Olea and PerfusionGo showed similar agreement, and it was higher than that reported in previous studies (Xiong et al., 2019). Therefore, we believe that Olea and PerfusionGo had the same capacity in accurately diagnosing and classifying patients with infarct volume <70 ml, which may have important practice implications.

This study has some limitations. First, given the retrospective study design, we were unable to precisely control for the time between CTP and recanalization, as well as the time between CTP and follow-up imaging acquisition, and make these factors consistent in all patients. Some patients did not receive recanalization treatment immediately after CTP, and the volume of the IC continued to expand. To reduce these confounding factors, we attempted to enroll patients who underwent successful recanalization and been excluded from the CTP to recanalization period of 4 h. Second, this study currently only focused on AIS patients with occlusion of intracranial anterior-circulation large vessels. In the future, we aim to assess a larger and more inclusive cohort of patients. Third, the final imaging examination used to estimate FIV was CT in a subset of patients who were unable to undergo DWI. Although follow-up CT has been utilized as an effective tool for determining FIV, it is possible that this can result in discrepancies when compared to more accurate DWI (Inoue et al., 2014). Last, all patients we included were from a comprehensive stroke center, and this study did not include patients outside this institution, even though it is a comprehensive stroke center that receives a large number of stroke cases each year.

## 5. Conclusion

The two software we tested have differences in the evaluation of IC and penumbra. Olea tended to obtain larger IC and smaller penumbra than PerfusionGo. Olea’s predicted FIV was more closely correlated with the true FIV than PerfusionGo’s. However, neither software was sufficiently accurate in the satisfying prediction of FIV, hence, accurate assessment of infarction based on CTP post-processing software remains challenging. Our results may have important practice implications for the clinical use of perfusion post-processing software. Because current software that use thresholds to predict FIV are still inaccurate and have a non-negligible risk, we will attempt to predict the FIV and provide data for assistance to clinicians for the diagnosis and treatment via a deep-learning threshold-free approach in future studies.

## Data availability statement

The raw data supporting the conclusions of this article will be made available by the authors, without undue reservation.

## Ethics statement

The studies involving human participants were reviewed and approved by The First Affiliated Hospital of Chongqing Medical University. The patients/participants provided their written informed consent to participate in this study.

## Author contributions

JLiu, TL, JWa, and YL contributed to conception and design of the study. JLiu, JWu, SG, YY, and JLi organized the database. JLiu and JWa performed the statistical analysis. JLiu wrote the first draft of the manuscript. JLiu, JWa, and HR performed the editorial review of the manuscript. TL and YL guaranteed the integrity of the study. All authors contributed to the article and approved the submitted version.

## Funding

This study was funded by the Chongqing Talents Program Project of Technological Innovation and Application Development of Chongqing Science and Technology Bureau (cstc2022ycjh-bgzxm0230), the Key Project of Technological Innovation and Application Development of Chongqing Science and Technology Bureau (CSTC2021 jscx-gksb-N0008) and the Research Project of Health Committee of Hunan Province (202209013170).

## Conflict of interest

The authors declare that the research was conducted in the absence of any commercial or financial relationships that could be construed as a potential conflict of interest.

## Publisher’s note

All claims expressed in this article are solely those of the authors and do not necessarily represent those of their affiliated organizations, or those of the publisher, the editors and the reviewers. Any product that may be evaluated in this article, or claim that may be made by its manufacturer, is not guaranteed or endorsed by the publisher.
